# Transcriptional Response of *Pectobacterium carotovorum* to Cinnamaldehyde Treatment

**DOI:** 10.4014/jmb.2311.11043

**Published:** 2023-12-25

**Authors:** Jihye Jung, Dawon Jo, Soo-Jin Kim

**Affiliations:** Division of Agricultural Microbiology, National Institute of Agricultural Science, Rural Development Administration, Wanju 55365, Republic of Korea

**Keywords:** Cinnamaldehyde, *Pectobacterium* spp., Minimum Inhibitory Concentration (MIC), Minimum Bactericidal Concentration (MBC), nitrate reductase pathways

## Abstract

Cinnamaldehyde is a natural compound extracted from cinnamon bark essential oil, acclaimed for its versatile properties in both pharmaceutical and agricultural fields, including antimicrobial, antioxidant, and anticancer activities. Although potential of cinnamaldehyde against plant pathogenic bacteria like *Agrobacterium tumefaciens* and *Pseudomonas syringae* pv. *actinidiae* causative agents of crown gall and bacterial canker diseases, respectively has been documented, indepth studies into cinnamaldehyde’s broader influence on plant pathogenic bacteria are relatively unexplored. Particularly, *Pectobacterium* spp., gram-negative soil-borne pathogens, notoriously cause soft rot damage across a spectrum of plant families, emphasizing the urgency for effective treatments. Our investigation established that the Minimum Inhibitory Concentrations (MICs) of cinnamaldehyde against strains *P. odoriferum* JK2, *P. carotovorum* BP201601, and *P. versatile* MYP201603 were 250 μg/ml, 125 μg/ml, and 125 μg/ml, respectively. Concurrently, their Minimum Bactericidal Concentrations (MBCs) were found to be 500 μg/ml, 250 μg/ml, and 500 μg/ml, respectively. Using RNA-sequencing analysis, we identified 1,907 differentially expressed genes in *P. carotovorum* BP201601 treated with 500 μg/ml cinnamaldehyde. Notably, our results indicate that cinnamaldehyde upregulated nitrate reductase pathways while downregulating the citrate cycle, suggesting a potential disruption in the aerobic respiration system of *P. carotovorum* during cinnamaldehyde exposure. This study serves as a pioneering exploration of the transcriptional response of *P. carotovorum* to cinnamaldehyde, providing insights into the bactericidal mechanisms employed by cinnamaldehyde against this bacterium.

## Introduction

Cinnamaldehyde, a natural compound extracted from cinnamon bark essential oil, is receiving significant attention across various areas, most notably within the pharmaceutical and food industries, due to its multifaceted properties. These include antimicrobial, anti-inflammatory, antioxidant, anticancer, and anti-obesity activities [1-3]. Cinnamaldehyde has demonstrated potent antimicrobial effects against bacterial strains such as *Streptococcus mutans* [1], which is associated with the human oral cavity, and *Enterococcus faecalis*, known to colonize the urinary tract, cause endocarditis, and persist in root canal infections [4]. Its anti-inflammatory action, notably the effect of trans-cinnamaldehyde on lipopolysaccharide-stimulated macrophages, is attributed to the suppression of the phosphorylation of mitogen-activated protein kinases (MAPKs) [2]. Furthermore, cinnamaldehyde 's therapeutic potential extends to vascular smooth muscle cells (VSMCs), which are implicated in the development of atherosclerosis. In this context, cinnamaldehyde activates nuclear factor erythroid 2-related factor 2 (Nrf2) antioxidant enzymes and diminishes proliferation and intimal hyperplasia in Zucker Diabetic Fatty (ZDF) rats following balloon injury [5]. In the area of metabolic health, cinnamaldehyde 's preventive effects against diabetes and obesity are gaining attention, but understanding the underlying mechanisms remains an ongoing effort [3]. From a regulatory perspective, cinnamaldehyde has secured approval as a safe natural active compound from authorities such as the Food and Drug Administration (FDA) in the United States and Europe with a recommended daily intake of 1.25 mg/kg [3], cinnamaldehyde has found its place as a flavor additive in everyday chemicals and dietary supplement products.

In the field of agriculture, studies are ongoing regarding the antimicrobial properties of cinnamaldehyde against plant pathogens. It exerts antagonistic effects against fungi like *Aspergillus flavus* and *Phytophthora capsici*, making it a potent shield against post-harvest spoilage [6, 7]. Moreover, cinnamaldehyde strengthens the innate defenses of specific crops. For instance, post-harvest citrus fruits treated with cinnamaldehyde exhibit enhanced resistance to pathogens like *Pecicillium digitatum* and *Geotrichum citri-aurantii* which cause green mold and sour rot, respectively [8] This resilience is further correlated with increased enzymatic activities, namely phenylalanine ammonia-lyase (PAL), peroxidase (POD), polyphenol oxidase (PPO), β-1, 3-glucanase (GLU) and chitinase (CHT), which strengthening their defensive stance [8]. Recent discoveries have further highlighted cinnamaldehydés inhibitory effects on *Botrytis cineria* in cherry tomatoes and *Colletotrichum fructicola* in Chinese sorghum [9, 10]. Additionally, it exhibits distinct action on *Fusarium sambucinum* in potatoes, where it disrupts ergosterol biosynthesis, essential for cell membrane stability [11]. Expanding its range, cinnamaldehydés antimicrobial effect extends beyond fungi, exhibiting activity against both gram-positive and gram-negative bacteria. Within this spectrum, its actions against plant pathogens such as *Agrobacterium tumefaciens*, implicated in crown gall disease, and *Pseudomonas syringae* pv. *actinidiae*, responsible for bacterial canker disease, are particularly noteworthy [12, 13]. Preliminary studies offer promising insights into cinnamaldehydés antimicrobial potency; however, the exact mechanisms remain elusive [12, 13].

With this foundation, we examine cinnamaldehydés antimicrobial capacities against *Pectobacterium* spp. This bacterium, being facultative anaerobic, triggers soft rot disease in a wide range of hosts such as cabbage, potato, radish, carrot, tomato, cauliflower, bell pepper, and cucumber, leading to significant crop damage [14-16]. Soft rot disease can be indirectly controlled through methods such as water management and plant nutrition; however, as of today, complete control is difficult to achieve [17]. Since cinnamaldehyde is a natural compound, it would be suitable for use in several crops if it demonstrated antibacterial activity against *Pectobacterium* spp. By employing RNA-sequencing, we aim to unveil the antimicrobial mechanisms that cinnamaldehyde uses against *Pectobacterium*. Through this pursuit, we hope to enrich and potentially revolutionize sustainable crop protection paradigms with the use of the natural compound cinnamaldehyde.

## Materials and Methods

### Bacterial Strains and Growth Conditions

Strains of *Pectobacterium odoriferum* JK2, *P. carotovorum* BP201601, and *P. versatile* MYP201603 were obtained from Highland Agriculture Research Institute, Rural Development Administration. All *Pectobacterium* spp. were cultivated in Luria-Bertani (LB) medium (Difco Laboratories, USA) and incubated at 30°C for 24 h.

### Minimum Inhibitory Concentration and Minimum Bactericidal Concentration Test

We evaluated the minimum inhibitory concentration (MIC) and minimum bactericidal concentration (MBC) values of cinnamaldehyde against *P. carotovorum* spp. according to recommended procedure from the Clinical and Laboratory Standards Institute [18]. Cinnamaldehyde (Sigma-Aldrich) was prepared in 2% Ethyl alcohol (EtOH) and tested across a range of concentrations from 31.25 to 1000 μg/ml. We then introduced bacterial solutions (at a density of 1 × 10^6^ to 1 × 10^8^ CFU/ml) and allowed them to incubate at 30°C for a duration of 24 h. To ensure the validity of our experimental design, an EtOH control (consisting of the sterile culture medium and EtOH) was included. The MIC was identified as the minimum concentration at which no noticeable bacterial growth occurred. The MBC was subsequently established as the smallest concentration that prevented any bacterial colony formation upon subsequent cultivation on agar surfaces. All tests were conducted in triplicate during each session, and the findings presented are an aggregate of three independent experiments.

### RNA Preparation for RNA Sequencing

*P. carotovorum* BP201601 strain was treated with 500 μg/ml cinnamaldehyde or 2% EtOH(control) and subsequently incubated at 30°C in a shaking incubator. After an 8 h of treatment, cells were harvested by centrifugation at 8,000 ×*g* for 10 min, and bacterial pellets were stored in a -80°C deep freezer until RNA extraction. RNA was extracted using the Trizol reagent, the QIAzol Lysis Reagent, and the RNeasy Mini Kit (Qiagen, USA) following the manufacturer’s instructions. RNA samples were prepared with three biological replicates in each group. The total RNA concentration was determined using Quant-IT RiboGreen (Invitrogen, USA). The integrity of the total RNA was assessed using the TapeStation RNA screentape (Agilent Technologies, USA). For each sample, a library was prepared from 1 μg of total RNA using the Illumina TruSeq Stranded mRNA Sample Prep Kit (Illumina Inc., USA). Initially, bacterial rRNA-depleted samples were prepared with the NEBNext rRNA Depletion kit (NEB, USA). Post rRNA depletion, the residual RNA was fragmented using divalent cations at high temperatures.

### cDNA Synthesis and Library Construction and Annotation

The fragmented RNA was reverse transcribed into first-strand cDNA using SuperScript II reverse transcriptase (Invitrogen) and random primers. This step was followed by second-strand cDNA synthesis employing DNA Polymerase I, RNase H, and dUTP. The cDNA fragments underwent end repair, the addition of an 'A' base, and adapter ligation. The resultant products were purified and enriched with PCR, yielding the final cDNA library. Library concentrations were quantified using the KAPA Library Quantification kits (KAPA Biosystems, USA) for Illumina Sequencing platforms, as per the qPCR Quantification Protocol Guide (KAPA Biosystems), and validated with the TapeStation D1000 ScreenTape (Agilent Technologies, USA). Indexed libraries were sequenced on an Illumina NovaSeq (Illumina, Inc.) with paired-end (2×100 bp) sequencing by Macrogen Incorporated. The raw reads obtained from the sequencer were preprocessed to discard low-quality and adapter sequences. The refined reads were then aligned to the *P. carotovorum* subsp. *carotovorum* PCC21 genome using Bowtie 1.1.2, an ultrafast, memory-efficient short read aligner leveraging the Burrows-Wheeler index and a quality-aware backtracking algorithm. Reference genome sequences and gene annotations were sourced from the NCBI Genome assembly and the NCBI RefSeq database, respectively. Post-alignment, HTSeq v0.10.0 was utilized to assemble the aligned reads into transcripts and quantify their abundance. Both gene-level and transcript-level quantifications were expressed in terms of raw read count, FPKM (Fragments Per Kilobase of transcript per Million mapped reads), and TPM (Transcripts Per Million).

### Analysis of Differentially Expression Genes (DEGs) and Functional Annotation

The relative abundances of genes were quantified in Read Count using StringTie. We conducted statistical analyses to identify differentially expressed genes (DEGs), utilizing estimates of abundance for each gene across samples with Read Count values of zero in more than one sample were excluded from analysis. To facilitate log2 transformation, 1 was added to the Read Count value of each filtered gene. The filtered data underwent log2 transformation and were then subjected to Relative Log Expression (RLE) normalization. The statistical significance of differential gene expression was determined using the nbinomWaldTest within the DESeq2 package, examining fold changes under the null hypothesis that there were no differences among the groups. The False Discovery Rate (FDR) was controlled by adjusting the *p*-values using the Benjamini-Hochberg algorithm. For the set of DEGs, hierarchical clustering analysis was carried out using complete linkage with Euclidean distance as a measure of similarity. Gene enrichment, functional annotation analysis, and pathway analysis for the significant gene list were conducted based on the blastGO platform (http://geneontology.org/) and the KEGG pathway database (https://www.genome.jp/kegg/).

### Hierarchical Clustering

Hierarchical clustering analysis was conducted using complete linkage with Euclidean distance as a measure of similarity. This was utilized to showcase the expression patterns of differentially expressed transcripts that met criteria of |fold change| ≥2 and raw *p* < 0.05.

### Volcano Plot

We utilized a volcano plot to visualize the differences between groups, plotting the log2 fold change (X-axis) against the -log10 *p*-value (Y-axis) obtained from the average comparisons of each group. All data analysis and visualization of differentially expressed genes were conducted using R version 4.2.2 (www.r-project.org).

### Expression Analysis Using qRT-PCR

The synthesis of first-strand cDNA for qRT-PCR was carried out using 1 μg of total RNA and SuperScript II reverse transcriptase (Invitrogen), following the protocol provided by the manufacturer. The expression of genes was analyzed using the CFX96 Real-time PCR detection system and SYBR green fluorescent dye (Bio-Rad). The PCR program included an initial denaturation at 95°C for 5 min, followed by 40 cycles of denaturation at 95°C for 15 sec, annealing at 57°C for 30 sec, and extension at 72°C for 30 sec. We used the 16S rRNA gene from *P. carotovorum* BP201601 as an internal control to normalize gene expression. Gene expression levels were calculated using the ΔΔCt method, and to facilitate comparison, we expressed them as relative values against the expression levels on Control or cinnamaldehyde. The specific sequences of the primers used are provided in Table S3.

### Data Availability Statement

The whole transcriptome sequence raw data were deposited in the NCBI database (https://www.ncbi.nlm.nih.gov/sra) with accession numbers SAMN38089555 and SAMN38089557 for Control and cinnamaldehyde treated samples, respectively.

## Results

### Antibacterial activity of Cinnamaldehyde against *Pectobacterium* spp.

**Fig. 1:**
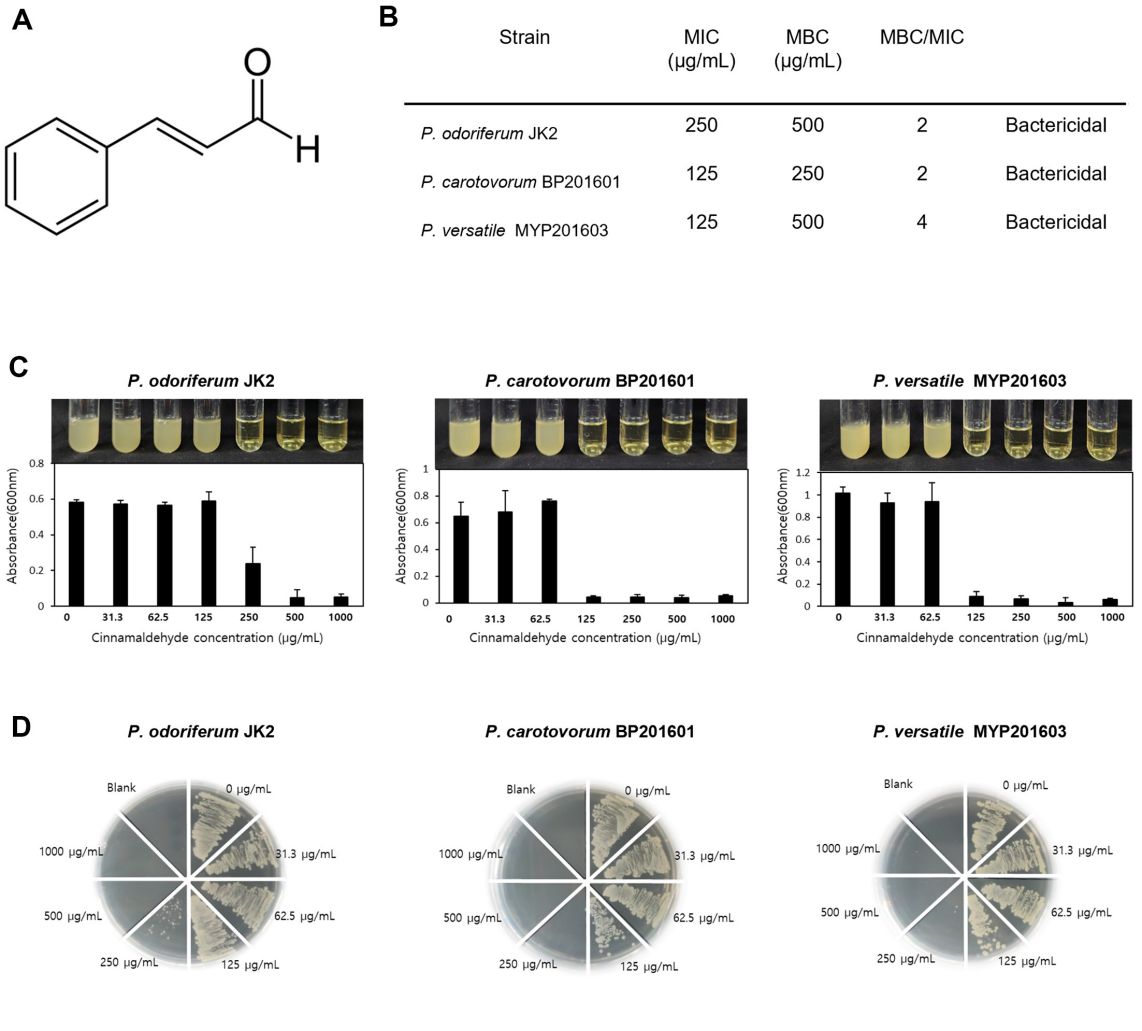
Bactericidal activity of Cinnamaldehyde against *Pectobacterium* spp. (**A**) Chemical structure of cinnamaldehyde. (**B**) MIC and MBC value of *P. odoriferum* JK2, *P. carotovorum* BP201601, *P. versatile* MYP201603 against cinnamaldehyde. (**C**) inhibition of growth of *P. odoriferum* JK2, *P. carotovorum* BP201601, *P. versatile* MYP201603 by cinnamaldehyde at the given concentrations was measured in LB broth after 24 h of incubation at 30°C. Bacterial turbidity was measured at OD = 600 nm, and present as the mean ± SE of three independent experiments. (**D**) Bactericidal concentrations of cinnamaldehyde for *Pectobacterium* spp. were determined by inoculating on agar plate from each replicates in broth dilution plate that shows a complete absence of growth and was incubated at 30°C for 24 h.

*Pectobacterium odoriferum* JK2, *P. carotovorum* BP201601, and *P. versatile* MYP201603 were isolated from various regions in Korea (Gangneung, Boseong, Miryang) and from different hosts (potato or cabbage). The genome sequences of these three *Pectobacterium* strains have also been deposited in the NCBI database (*P. carotovorum* JK2 - CP034938.1, *P. carotovorum* BP201601 - CP034236.1, *P. odoriferum* MYP201603 - CP051628.1). We tested the antimicrobial activity of cinnamaldehyde (Fig. 1A) against these three *Pectobacterium* strains as part of our approach for further transcriptome analysis.

The MIC values were determined to be 250 μg/ml for *P. odoriferum* JK2 and 125 μg/ml for both *P. carotovorum* BP201601 and *P. versatile* MYP201603 (Fig. 1B and 1C). In contrast, MBC values were found to be 500 μg/ml, 250 μg/ml, and 500 μg/ml for *P. odoriferum* JK2, *P. carotovorum* BP201601, and *P. versatile* MYP201603, respectively (Fig. 1B and 1D). The resulting MBC:MIC ratios suggest a bactericidal effect of cinnamaldehyde on all three *Pectobacterium* spp. strains. This conclusion is supported by the general consensus that an MBC to MIC ratio ≤ 4 typically indicates bactericidal activity [19].

To assess the bactericidal potency of cinnamaldehyde against densely cultured *Pectobacterium* spp., bacterial concentrations between 1 × 10^7^ CFU/ml and 1 × 10^8^ CFU/ml were exposed to either 125 μg/ml or 500 μg/ml of cinnamaldehyde. Within 8 h, the growth of *P. carotovorum* BP201601 was significantly inhibited by a concentration of 500 μg/ml cinnamaldehyde, whereas the 125 μg/ml concentration proved ineffective for cultures at 1 × 10^8^ CFU/ml (Fig. S1A and S1B). Additionally, 24 h after treatment with 500 μg/ml of cinnamaldehyde, bactericidal effects on 1 × 10^8^ CFU/ml cultures were evident in both *P. odoriferum* JK2 and *P. versatile* MYP201603 (Fig. S1A). Furthermore, at both 125 μg/ml and 500 μg/ml concentrations of cinnamaldehyde, all three bacterial strains demonstrated susceptibility when cultured at 1 × 10^7^ CFU/ml, showing effects as early as 4 h post-exposure (Fig. S1A and S1B).

### RNA Sequencing and Mapping Analysis

To unravel the mechanism underlying cinnamaldehyde’s bactericidal activity, we chose *P. carotovorum* BP201601, as it showed the lowest MBC value among the three tested *Pectobacterium* spp. (Fig. 1B). Cinnamaldehyde at 500 μg/ml has been observed to have bactericidal activity at densely cultured concentrations (1 × 10^8^ CFU/ml)(Fig. S1A). Consequently, *P. carotovorum* BP201601 was exposed to this concentration of cinnamaldehyde, known to exert bactericidal effects, and samples were collected at the 8-h mark for RNA sequencing.

Each sample library yielded between 21,826,458 to 36,637,298 raw reads. After trimming, the read counts ranged from 7,247,982 to 36,287,172, with Q20 > 98.74% and Q30 > 95.75% for each library (Table S1). These trimmed reads were aligned to the reference genome *P. carotovorum* BP201601.1 (GCF_009931195.1) using the Bowtie aligner. The alignment revealed that 63.34-95.48% of reads matched the reference genome (Table S2). Reads suppressed due to multiple mappings accounted for 0.17-0.53%, and 4.26-36.44% of the reads failed to align (Table S2).

### Analysis of Differentially Expressed Genes (DEGs)

**Fig. 2:**
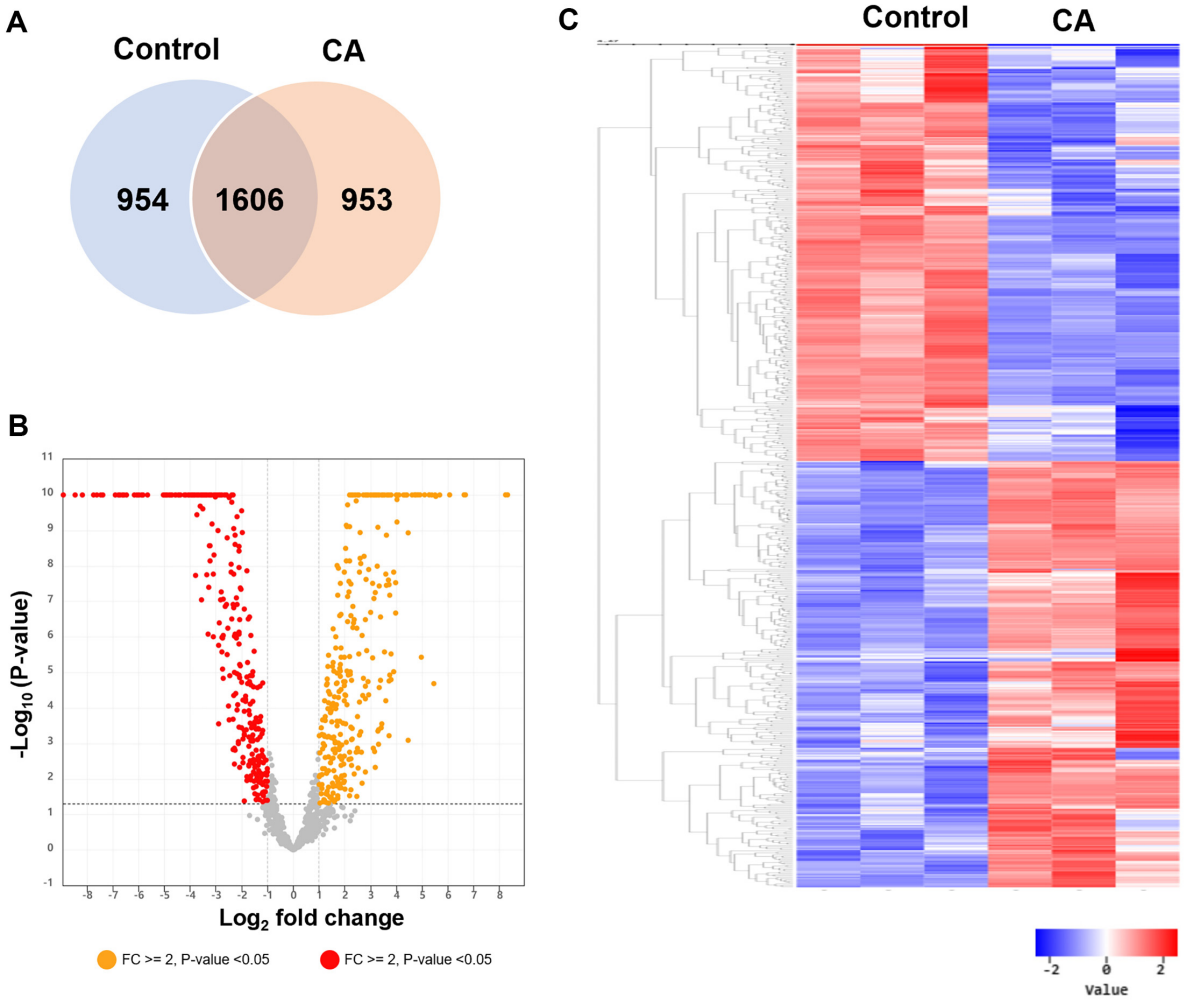
Differentially expressed genes (DEGs) in *P. carotovorum* BP201601 following cinnamaldehyde treatment. (**A**) Venn diagram showing the overlap of DEGs between the cinnamaldehyde (CA)-treated and untreated (Control) groups. (**B**) Volcano plot displaying DEGs in *P. carotovorum* BP201601 after cinnamaldehyde treatment. Genes with a log2 fold change of ≥2 or ≤−2 and a *p*-value of <0.05 are classified as DEGs. Up- and down-regulated DEGs are represented by red and yellow dots, respectively. (**C**) Heat map illustrating hierarchical clustering based on 1,907 DEGs with a log2 fold change of ≥2 or ≤−2 and a *p*-value of <0.05. Red and blue colors indicate up- and down-regulated DEGs in the cinnamaldehyde-treated group compared to the untreated group, respectively.

Upon comparing the gene expressions of cinnamaldehyde treated and untreated *P. carotovorum* BP201601, we identified a total of 1,907 DEGs. Of these, 953 genes were upregulated, while 954 were downregulated (Fig. 2A). The distribution and significance of these DEGs were visualized through a volcano plot and a hierarchical clustering heatmap (Fig. 2B and 2C). Annotation of the 1,907 DEGs using the NCBI-nr database highlighted that genes associated with nitrate reductase were distinctly upregulated in the cinnamaldehyde treated group (Supplementary S1). This suggests potential metabolic shifts in *P. carotovorum* in response to cinnamaldehyde exposure. A detailed list of all DEGs can be found in Supplementary S1. Overall, the data underscores the pronounced transcriptional adjustments of *P. carotovorum* BP201601 upon cinnamaldehyde treatment.

### Gene Ontology (GO) Annotation and KEGG Analysis of DEGs

**Fig. 3:**
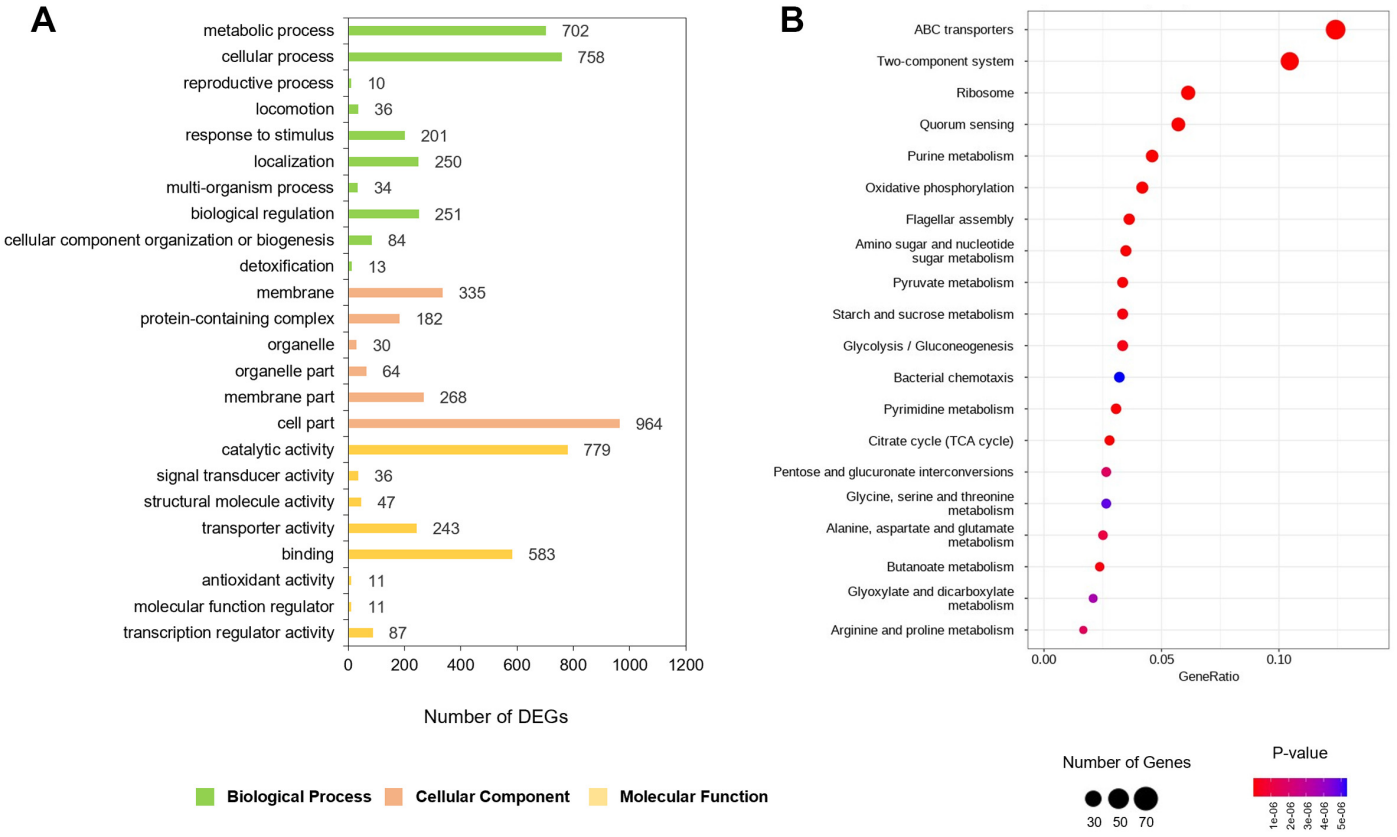
Gene Ontology (GO) enrichment and Kyoto Encyclopedia of Genes and Genomes (KEGG) pathway enrichment analysis of 1,907 DEGs. (**A**) GO annotation analysis of the 1,907 DEGs was carried out using the blastGO platform. The figure presents 24 identified GO terms from the three primary categories: “cellular components,” “molecular function,” and “biological process.” The numbers of up- and down-regulated DEGs in each identified GO term are also shown. (**B**) KEGG pathway enrichment analysis of the 1,907 DEGs was conducted using KEGG pathway database. The top 20 enriched pathways were determined based on *p*-value, rich factor, and the count of enriched genes.

GO labeling represents a globally accepted gene function classification system, comprised of three primary sectors: biological process (BP), cellular component (CC), and molecular function (MF). To explore the functional implications of the most pronounced up- or downregulated genes, we conducted a GO enrichment assessment based on the Fisher’s exact test, using a p value threshold of ≤ 0.05. Summarily, the DEGs were distributed across 47 GO terms: 21 under BP, 14 under CC, and 12 under MF. As depicted in Fig. 3A and Supplementary File 2, the predominant terms within BP include “cellular process (GO:0009987)”, “metabolic process (GO:0008152)”, and “biological regulation (GO:0065007)”, with respective DEG counts of 758, 702, and 251. Within the CC part, “cell part (GO:0044464)” “membrane (GO:0016020)”, “membrane part (GO:0044425)” with DEG counts of 964, 335, and 268, respectively (Fig. 3A and Supplementary S2). Within MF, the terms “catalytic activity (GO:0003824)”, “binding (GO:0005488)”, and “transporter activity (GO:0005215)” stood out, with corresponding DEG counts of 779, 583, and 243 (Fig. 3A and Supplementary S2).

To further understand the pathways potentially altered by cinnamaldehydés impact on *P. carotovorum* BP201601 growth, we mapped DEGs to the Kyoto Encyclopedia of Genes and Genomes (KEGG) database, performing a pathway enrichment analysis. This resulted in the identification of thirty significantly enriched pathways (bonferroni value ≤ 0.05) (Supplementary S3). These included pathways such as “Two-component system”, which plays a crucial role in bacterial signal transduction [20]; the “Ribosome”, essential for protein synthesis [21]; the “ABC transporters”, vital for nutrient uptake and drug resistance [22]; and the “Oxidative phosphorylation”, a key component of energy metabolism [23]. In our analysis of the top 20 KEGG pathways (Fig. 3B), we found that fourteen pathways – "purine metabolism," "oxidative phosphorylation," "amino sugar and nucleotide sugar metabolism," "pyruvate metabolism," "starch and sucrose metabolism," "glycolysis/gluconeogenesis," "pyrimidine metabolism," "citrate cycle," "pentose and glucoronate interconversions," "glycine, serine and threonine metabolism," "alanine, aspartate and glutamate metabolism," "butanoate metabolism," "glyoxylate and dicarboxylate metabolism," and "arginine and proline metabolism" are involved in metabolic pathways. These results indicate that cinnamaldehyde induces significant metabolic changes in *P. carotovorum* BP201601. Detailed representation and specifics of these enriched pathways can be referenced in Supplementary File S3.

### Exploring the Impact of Cinnamaldehyde on Metabolic Pathways in *P. carotovorum*

In the top 20 up-regulated DEGs, genes associated with nitrogen-reductase, specifically *gene-EH203_10035*, *gene-EH203_10045* (*narJ*), *gene-EH203_10040* (*narH*), *gene-EH203_09350* (*napA*), and *gene-EH203_09335*, exhibited notable increases in expression levels in cinnamaldehyde-treated samples, with respective fold changes of 297.4, 173.45, 109.93, 79.45, and 60.13, when compared to the control (Supplementary S1). Given the recognized upregulation of the *narKGHJI* gene cluster under oxygen deprivation, our attention gravitated towards metabolic pathways activated in hypoxic conditions [24].

**Fig. 4:**
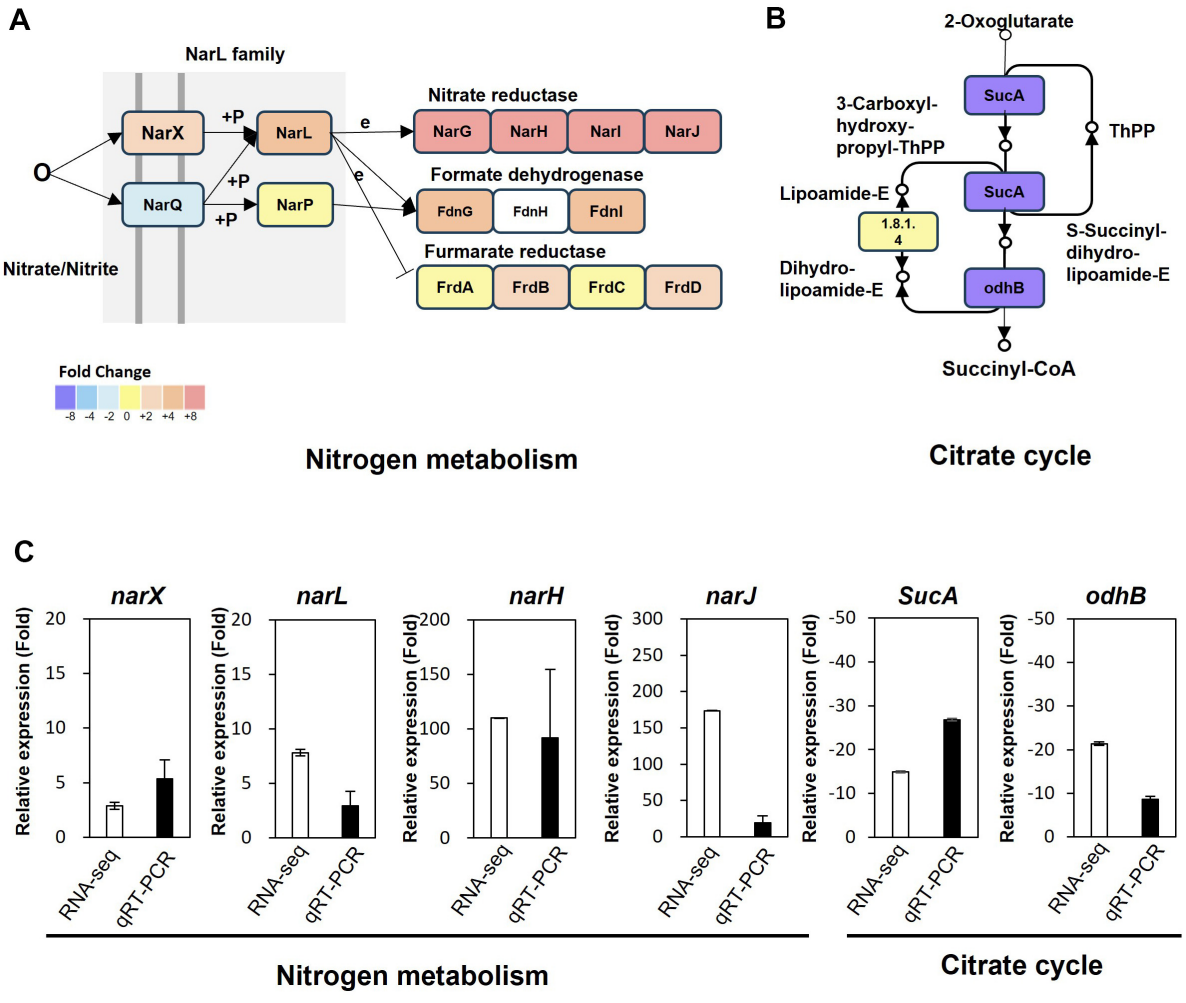
Pathways highlighting the differentially expressed genes identified in this study. (**A**) Up-regulated genes in the nitrogen metabolism pathway and. (**B**) Down-regulated genes in the citrate cycle pathway in RNA-seq. The scale represents the average fold change (FC) values between samples treated with cinnamaldehyde and the controls. The gene depicted in this figure are detailed in Supplementary File 4. (**C**) The gene expression data from RNA-seq and qRT-PCR validation of genes involved in the nitrate metabolism and citrate cycle pathways are shown, with relative values compared against the expression levels in the control. Error bars represent the standard error of the mean from three biological replicates.

Within the "nitrogen metabolism" pathway, genes such as *gene-EH203_10025* (*narX*), *gene-EH203_10020* (*narL*), *gene-EH203_10035* (*narG*), *gene-EH203_10040* (*narH*), *gene-EH203_10050* (*narI*), and *gene-EH203_10045* (*narJ*) involved in nitrate reductase functions, demonstrated a marked upregulation in cinnamaldehyde-treated samples (Supplementary S1 and Fig. 4A). Additionally, other pathways activated under oxygen-deficient conditions, including formate dehydrogenase N (FDH-N, subunit FdnGHI) with genes *gene-EH203_14760* (*fdnG*), *gene-EH203_14770* (*fdnI*), and fumarate reductase genes *gene-EH203_02410* (*frdB*), *gene-EH203_02400* (*frdD*), were significantly activated post cinnamaldehyde treatment [25].

The “citrate cycle" is pivotal for cellular energy generation in aerobic conditions. Intriguingly, cinnamaldehyde exposure led to a pronounced downregulation of the genes *gene-EH203_15330* (*sucA*) and *gene-EH203_15325* (*odhB*) (Supplementary S1 and Fig. 4B). Both *sucA* and *odhB* have integral roles within the citrate cycle, mediating the conversion of succinyl-CoA to succinate and the decarboxylation of 2-oxoglutarate to succinyl-CoA and CO2, respectively [26]. This evident downregulation may signal a cinnamaldehyde induced disturbance in aerobic cellular respiration. The expression of genes involved in nitrate reductase pathway and citrate cycle were also validated using qRT-PCR (Fig. 4C).

## Discussion

*P. carotovorum* spp., a gram-negative facultative anaerobic bacterium, presents a significant challenge in agriculture due to its pathogenicity. The unearthing of cinnamaldehydés antibacterial efficacy against this bacterium, as underscored by our study, offers a promising alternative for combating bacterial-induced plant diseases. Notably, cinnamaldehydés influence extends beyond simple bactericidal action, especially evident at higher bacterial concentrations.

In environments infested with bacteria, as seen in our experiments with densities reaching 1 × 10^8^ CFU/ml, cinnamaldehyde demonstrated bactericidal action against multiple strains of *Pectobacterium* spp. (Fig. S1). Our RNA sequencing revealed a pronounced metabolic shift, notably the increase in nitrate reductase genes, highlighting the bacterium's adaptability. While cinnamaldehyde seems to inhibit aerobic respiration by targeting citrate cycle genes such as *sucA* and *odhB*, the bacteria appear to adapt to this condition by upregulating anaerobic pathways, specifically nitrate reduction [28, 29]. Indeed, Nitrate metabolism is an adaptation mechanism used by many bacteria for survival in anaerobic environments [28]. Indeed, many other bacteria such as *Bacillus subtilis*, *Actinobacillus pleuropneumoniae*, utilize nitrate metabolism under anaerobic conditions showing increased *nitrate reductase* (*Nar*) gene expression [27, 28]. Furthermore, under hypoxic conditions where lipids might act as an alternate energy substrate replacing oxygen, the "Lipoic acid metabolism" pathway genes, *gene-EH203_15675* (*LipB*) and *gene-EH203_15680* (*LipA*), also showed enhanced expression in cinnamaldehyde treated samples (Supplementary File 1 and Fig. S2). The heightened emphasis on lipoic acid metabolism pathways seems to underscore a shift towards anaerobic metabolism (Fig. S2) [30], especially in the face of cinnamaldehyde-induced oxygen deprivation condition.

In other mechanisms studies, bactericidal actions of cinnamaldehyde against *Listeria monocytogenes* suggested inhibition on energy generation, specifically inhibition of glucose uptake or utilization, and effect membrane permeabilities [31]. Similarly, top 20 KEGG pathway analysis in our study, cinnamaldehyde treated *P. carotovorum* BP201601 showed 89, 75 DEGs on “ABC transporters” and “Two-component system”, respectively. Which are related in glucose uptake and membrane permeability [32, 33].

Quorum sensing is defined as cell-to-cell communications that occurs through the secretion of autoinducers when bacterial populations reach a threshold level. In previous studies, cinnamaldehyde has been shown to have anti-quorum sensing activities in both human and plant pathogenic bacteria, such as *Aeromonas hydrophila*, *Pseudomonas aeruginosa*, *P. fluorescence*, *A.tumefaciens* [34-37]. Quorum sensing plays a vital role in bacterial virulence, such as biofilm formation. Anti-quorum sensing strategies, therefore, could serve as effective anti-infective approaches. In our studies, we observed significant changes in 41 DEGs related to quorum sensing pathways upon cinnamaldehyde exposure (Supplementary S3).

Cinnamaldehyde is also suggested promising molecules in pharmaceutical area. As their bactericidal effect on pathogenic E.coli strain 042 which cause diarrhea, cinnamaldehyde-treated mice showed lower-levels of colonization by than the untreated groups [38]. In pathway studies, cinnamaldehyde inhibits the synthesis of RNA, DNA and protein leading to bacteria to total collapse [38]. Similarly, In metabolomics studies using LC-MS, cinnamaldehyde changed E.coli metabolism through interactions with proteins, nucleic acids, lipids, and carbohydrates [39]. In our studies, KEGG pathway on DEGs showed “Purine metabolism”, “Pyrimidine metabolism”, it is a crucial component for RNA and DNA, and “Ribosome”, “Alanine, aspartate and glutamate metabolism”, and “Arginine and proline metabolism” which are related in protein synthesis. Similar to previous studies [38, 39], cinnamaldehyde may also effect on RNA, DNA, and protein synthesis in *P.carotovorum* BP201601, however, the studies of exact mechanisms are needed for further studies.

The antibacterial activity through metabolic change presents a paradigm shift from traditional antibiotics. Unlike many conventional antimicrobial agents that target specific bacterial functions or structures [40, 41], cinnamaldehyde seems to impose a metabolic stress, pushing bacteria towards potentially less efficient or unsustainable metabolic pathways. While the bacteria attempt to adapt, the consequent over-reliance on these pathways might create an unsustainable intracellular environment, leading to their eventual downfall. This mode of action offers a compelling advantage in the contemporary battle against antibiotic resistance. Many bacterial strains, having been exposed to conventional antibiotics, have developed multi-drug resistance (MDR) [42]. For example, streptomycin has been considered to control soft rot disease in agriculture [43], however, streptomycin-resistant *Pectobacterium* strains have also been identified [44]. Therefore, the use of antibiotics, especially in open fields, is very dangerous because it accelerates the development of bacteria resistance. Cinnamaldehyde, with its unique mode of action that redirects bacterial metabolism rather than directly targeting growth, might be less prone to engender resistance, positioning it as a potential alternative or adjunct to traditional antibiotics [45]. Moreover, if cinnamaldehyde proves to be environmentally stable, its application could extend beyond targeted treatments to broad-spectrum agricultural pest management, possibly with minimal environmental impacts. Its potential impacts on bacterial virulence factor production, and efficacy against a broader spectrum of phytopathogens, if explored further, could establish cinnamaldehyde as a cornerstone in sustainable agricultural strategies. Future endeavors should delve deeper into cinnamaldehydés precise intracellular targets, further elucidating its unique mode of action. In an era where MDR is increasingly challenging the effectiveness of established antimicrobials, cinnamaldehyde emerges not just as an alternative but as an exemplar of how we might rethink our approach to bacterial pathogens.

## Supplemental Materials

Supplementary data for this paper are available on-line only at http://jmb.or.kr.
