# Supplementary material for: Transcriptional Response of Pectobacterium carotovorum to Cinnamaldehyde Treatment
Source: J Microbiol Biotechnol. 2023 Dec 25;34(3):538–46. doi: 10.4014/jmb.2311.11043 (PMC11016793; doi:10.4014/jmb.2311.11043)
Supplement: Supplementary file 1 [file jmb-34-3-538-supple.pdf]

J. Microbiol. Biotechnol. 2024. 34(3): 538–546

https://doi.org/10.4014/jmb.2311.11043

Supplementary Table and Figure

Table S1. Characteristics of the reads from Cinnamaldehyde treated or untreated libraries in *P. carotovorum* BP201601.

Sample id	Raw Data		Trimmed Data		GC(%)	Q20(%)	Q30(%)
	Total read bases	Total reads	Total read bases	Total reads			
Control_1	2,477,476,066	24,529,466	2,442,723,772	24,285,292	46.48	99.09	96.65
Control_4	2,204,472,258	21,826,458	2,174,337,119	21,610,870	45	99.08	96.57
Control_5	3,700,367,098	36,637,298	3,649,263,753	36,287,172	47.07	99.02	96.36
CA_500_3	2,493,741,308	24,690,508	2,171,545,627	21,799,246	47.6	98.95	96.22
CA_500_4	2,360,851,164	23,374,764	1,955,194,234	19,778,542	48.18	98.96	96.22
CA_500_5	3,111,102,798	30,802,998	705,590,814	7,247,982	44.16	98.74	95.75

Table S2. Summary of RNA-seq alignment.

Sample ID	Processed reads	Mapped reads	Failed to align reads	Multiplemapped reads
Control_1	24,285,292	23,123,778 (95.22%)	1,116,866 (4.60%)	44,648 (0.18%)
Control_4	21,610,870	20,634,372 (95.48%)	919,922 (4.26%)	56,576 (0.26%)
Control_5	36,287,172	34,334,400 (94.62%)	1,890,938 (5.21%)	61,834 (0.17%)
CA_500_3	21,799,246	17,843,922 (81.86%)	3,838,866 (17.61%)	116,458 (0.53%)
CA_500_4	19,778,542	15,136,864 (76.53%)	4,546,598 (22.99%)	95,080 (0.48%)
CA_500_5	7,247,982	4,591,230 (63.34%)	2,641,490 (36.44%)	15,262 (0.21%)

Table S3. Primers used in this study.

Gene	Forwards	Reverse
16S rRNA	GGCAGCAGTGGGGAATATTG	CTTTACGCCCAGTCATTCCG
narX	GATGCCGATCCTGAACGAAC	CGCCATATTGCCCGTGTTTA
narL	GGACCCGGATTTGATTCTGC	GCAGCCAGTAAATCTTCCGG
narH	TGAAACGTATGCTGGCGATG	TGAATTTGGTATCGCTGCCG
narJ	GACTACCTGCCGCTGTATCT	ACCTGTTCCTCTTCCCACAC
sucA	GACTCCGTGCTATCCGAAGA	CTCTGGACCTTGACCTTCGT
odhB	GCGTCTTTGGCTCTCTGATG	TCCAGCATCTCTTTCACCGT

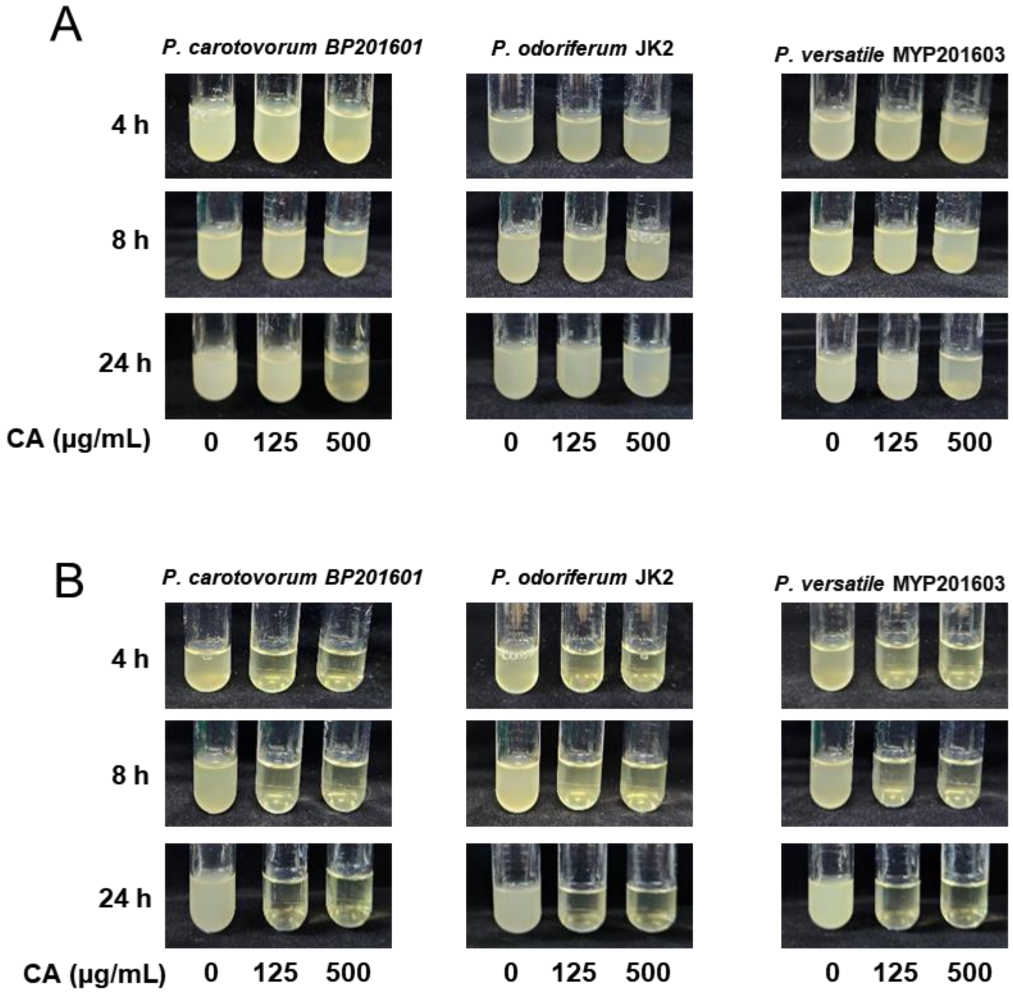

Supplementary Fig. S1. Bactericidal effect of cinnamaldehyde on *Pectobacterium* spp. bactericidal effect of cinnamaldehyde on *P. carotovorum* BP201601, *P. odoriferum* JK2 and *P.versatile* MYP201603 based on different concentration values of 1 × 10^8 CFU/mL **(A)** and 1 × 10^7 CFU/mL **(B)**.

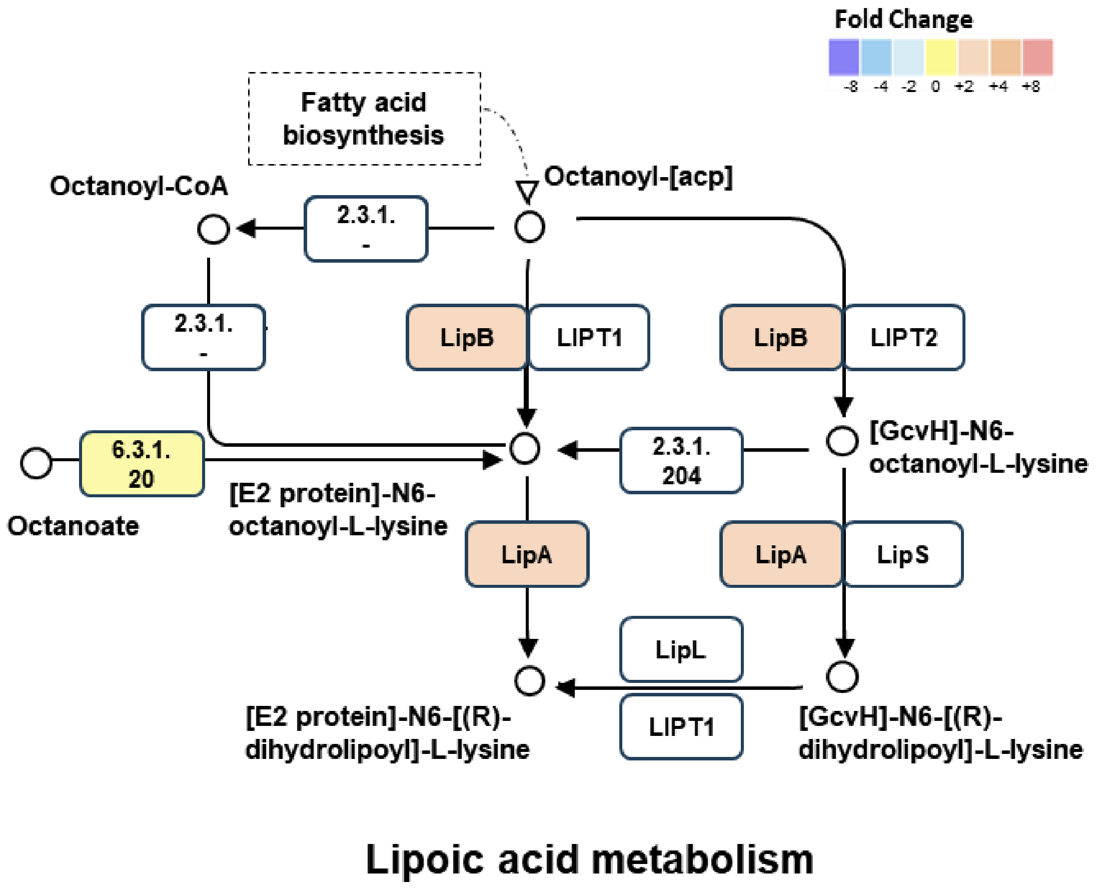

Supplementary Fig. 2. Pathways highlighting the differentially expressed genes identified in this study. Up-regulated genes in the “Lipoic acid metabolism” pathway in RNA-seq. The scale represents the average fold change (FC) values between samples treated with cinnamaldehyde and the controls. The gene depicted in this figure are detailed in Supplementary File4.
